# Unveiling the dynamic interplay between the hub- and spoke-components of the brain's semantic system and its impact on human behaviour

**DOI:** 10.1016/j.neuroimage.2019.05.059

**Published:** 2019-10-01

**Authors:** Rocco Chiou, Matthew A. Lambon Ralph

**Affiliations:** MRC Cognition and Brain Sciences Unit, University of Cambridge, UK

**Keywords:** Semantic memory, Hub-and-spoke, Anterior temporal lobe, Connectivity, Object recognition

## Abstract

The neural architecture of semantic knowledge comprises two key structures: (*i*) A set of widely dispersed regions, located adjacent to the sensorimotor cortices, serve as *spokes* that represent various modality-specific and context-dependent contents. (*ii*) The anterior-temporal lobe (ATL) serves as a *hub* that computes the nonlinear mappings required to transform modality-specific information into pan-modality, multifaceted concepts. Little is understood regarding whether neural dynamics between the hub and spokes might flexibly alter depending on the nature of a concept and how it impinges upon behaviour. Using fMRI, we demonstrate for the first time that the ATL serves as a ‘pivot’ which dynamically forms flexible long-range networks with cortical modules specialised for different domains (in the present case, the knowledge about actions and places). In two experiments, we manipulated semantic congruity and asked participants to recognise visually presented items. In Experiment 1 (dual-object displays), the ATL increased its functional coupling with the bilateral frontoparietal action-sensitive system when the objects formed a pair that permitted semantically meaningful action. In Experiment 2 (objects embedded in a scene), the ATL augmented its coupling with the retrosplenial cortex of the place-sensitive system when the objects and scene formed a semantically coherent ensemble. Causative connectivity revealed that, while communication between the hub and spokes was bidirectional, the hub's directional impact on spokes dwarfed the strength of the inverse spoke-to-hub connectivity. Furthermore, the size of behavioural congruity effects co-varied with the strength of neural coupling between the ATL hub and action- / place-related spokes, evident both at the *within*-individual level (the behavioural fluctuation across scanning runs) and *between*-individual level (the behavioural variation of between participants). Together, these findings have important implications for understanding the machinery that links neural dynamics with semantic cognition.

## Highlights

• Object recognition recruits the anterior temporal lobe (ATL), regardless of tasks and stimuli
• Processing semantic congruity about action and place recruits respective domain-specific regions
• Neural dynamics between the ATL and action/place-specific regions co-varies with behavioural congruity effects
• Semantic knowledge requires a division of labour between domain-general (ATL) and domain-specific regions

## 1 Introduction

The neurobiological substrates of semantic knowledge have always been the primary focus of cognitive neuroscience. A prominent theory fractionates the neural underpinning of semantic knowledge into two key constituents: the anterior-temporal lobe (ATL) as the *hub* and multiple modality- or category-specific regions as the *spokes* (Lambon Ralph, Jefferies, Patterson and Rogers, 2017; Patterson and Lambon Ralph, 2015; Patterson et al., 2007; Rogers et al., 2004). In this hub-and-spoke theory, the ATL hub integrates information emanated from multiple modality- or content-specific spokes to generate a coherent concept. For instance, the semantic attributes of a steering-wheel comprise its motoric attribute (to rotate), its locative attribute (inside a vehicle), and visual attribute (circular in shape). Such modality- or feature-specific information is hypothesised to be handled by cortical ‘spokes’ specialised in processing action, place, and shape, respectively. Over and above these fragments of semantic information, the ATL is assumed to aggregate neural processing from different sources and enable the formation of a unified concept about ‘steering-wheel’. This theory provides an important ‘hybrid middle-ground’ between contrasting theoretical views: It acknowledges the prominence of sensory-motoric information as building blocks of semantic knowledge, refuting the extreme amodal view that assumes semantic knowledge is purely propositional and symbolic (e.g., Fodor, 1983). However, its proposed ‘hub’ component enables this theory to offer explanations for a plethora of observations that the embodied cognition theory struggles to tackle (for discussion about the insufficiency of strong embodied cognition views, see Lambon Ralph, 2014; Lambon Ralph et al., 2017).

Since its genesis, the hub-and-spoke theory has been continually updated as neuroimaging techniques advance. Initially, this theory was built upon contrastive patterns of behavioural deficits from different types of neurological patients. Whereas patients with semantic dementia (SD) have focal atrophy of the ATL hub and show semantic deficits that extend over various conceptual domains and input modalities, patients with agnosia or apraxia have lesions circumscribed to the typical spoke regions and show selective impairments in certain domains (for review, see Hodges and Patterson, 2007; Reilly et al., 2010). Later, with the advent of effective neuroimaging protocols that surmount signal dropout at the basal part of the brain, researchers have begun to observe robust activation of the ATL when healthy participants perform semantic tasks on words, pictures, and environmental sounds, supporting this region's pan-modality nature (e.g., Binney et al., 2010; Chiou et al., 2018; Reilly et al.,2016a; Visser and Lambon Ralph, 2011). Apart from task-based fMRI data, diffusion-tensor imaging has revealed how white-matter tracts from multiple modality-specific regions converge at the ATL, providing the conduits for information exchange between the ATL and spokes (Bajada et al., 2017; Binney et al., 2012). Moreover, using lesion-symptom mapping techniques, researchers have shown that the putative ATL hub centres on the anterior section of the fusiform gyrus (Mion et al., 2010) and SD patients' severity of semantic deficits correlates both with the extent of ATL atrophy and the reduced connectivity between ATL hub and modality-specific spoke regions (Guo et al., 2013). Together, multiple threads of evidence consistently suggest the existence and necessity of a hub-and-spoke system in semantic cognition.

Our understanding about the relationship between the hub and spokes, nevertheless, is far from complete. There are three outstanding questions awaiting further investigation: *First*, there remains a paucity of evidence about whether the ATL flexibly cooperates with different spokes under different semantic contexts. Exploiting connectivity analysis holds promise for verifying the ATL's role as a functional hub with adaptive communication with other regions. *Second*, more definitive evidence is needed about whether the communication between hub and spoke has any functional impact on behaviour; while reduced structural connectivity between the hub and spokes correlates with patients' deterioration in performance, we are still agnostic about whether this holds true for healthy brains. *Third*, there is still a dearth of evidence about whether the communication between hub and spoke and is unidirectional (e.g., the hub dictates to spokes) or bidirectional, and (if it is bidirectional) whether the two-way communication is symmetrical (e.g., the two parties speak ‘equally loud’ to one another) or asymmetrical.

To answer these questions, we conducted two fMRI experiments to investigate the relationship between the ATL hub and functionally disparate spokes. We presented pictorial stimuli and probed action knowledge (Experiment 1) and place knowledge (Experiment 2). Previous investigations into the neural basis of action and place knowledge have revealed that these two forms of semantic concepts rely upon separable cortical structures: action knowledge recruits a set of frontoparietal regions well-established as the action-semantics system, including the inferior frontal gyrus, inferior/superior parietal lobules, and motor cortices (for review, see Ishibashi et al., 2016; van Elk et al., 2014). By contrast, place knowledge recruits a set of ventro-medial cortices known as the scene-processing system, including the parahippocampal cortex, lingual gyrus, precuneus, and retrosplenial cortex (for review, see Aminoff et al., 2013; Epstein, 2014). While prior studies have identified these separable functionally-specific systems for action and place knowledge, the role of the ATL has long been ignored due presumably to signal dropout (Visser et al., 2010) or a selective focus on the action- or place-specific regions. Hence, it remains unclear whether these apparently separable systems would be integrated by the ATL, and whether the extent of integration impinges upon behavioural performance. These questions directly put the hub-and-spoke hypothesis to the test – we investigated whether both action and place knowledge recruits the ATL-structure as a common nexus, as well as whether connectivity analysis would reveal any change in the alliance between the hub and spokes (the action- and scene-specific system) when participants retrieve different types of knowledge.

To pre-empt the main findings, we found that the ATL flexibly interacts with the action- or place-specific system in response to contexts that emphasise action affordance or object-background relationship. Moreover, we identified critical neural signatures that the extent to which performance is affected by semantic congruity of action/place is reflected in the strength of neural connectivity between the ATL and the action/place system. These data highlight the omnipresent involvement of the ATL in various contexts of object recognition and, more importantly, its pivotal role in cooperating with domain-specific modules to enable accurate identification and efficient performance.

## 2 Materials and method

### 2.1 Participants

Twenty native English-speaking volunteers (18 females, age range: 20–32) gave informed consent before participating in the fMRI experiments. All were right-handed (assessed using the Edinburgh Handedness Inventory), completed safety screening for MRI before participation, and reported no history of neurological disease/injury or psychiatric condition. The study was reviewed and approved by the university research ethics committee.

### 2.2 Experimental design

Participants completed two experiments in a single session. There were eight runs of scanning. In Scan 1–3 (Experiment 1), participants viewed displays of dual objects, and performed a task probing the influences of action-related semantics. In Scan 4–6 (Experiment 2), participants viewed objects imbedded within a scene, performed a task probing the influences of scene-related semantics. In Scan 7–8, they performed a conventional 1-back localiser task to identify object-sensitive voxels of the lateral occipital cortex (LOC). The localiser data were used for a separate study and not reported here.

We took several methodological issues into consideration when designing our experiments: First, to overcome the severe signal dropout in standard gradient-echo fMRI that particularly affects the ventral ATL we used a dual gradient-echo procedure that has been demonstrated to effectively and reliably improve signal acquisition at the fundus of the ATL (e.g., Halai et al., 2014; Jackson et al., 2015). Second, we sought to employ connectivity analysis, psychophysiological interaction (PPI) and dynamic causal modelling (DCM), to investigate task-dependent connectivity to the ATL. Compared to event-related design, block design offers a more conducive situation for task-induced changes in neural connectivity to unfold over time. This is particularly the case for PPI – it has been shown that PPI analysis tends to produce spurious null results and poor fit between haemodynamic and neural responses when it is conducted on event-related fMRI data (Gitelman et al., 2003; O’Reilly et al., 2012). By contrast, block design has been shown to enable more robust and accurate estimates of PPI connectivity (Cisler et al., 2014; Kim and Horwitz, 2008). Therefore, we opted to use a block design that optimised statistical power and modelling accuracy for connectivity analysis. Third, meta-analysis has shown that using an active, non-semantic task as baseline (e.g., perceptual judgements on scramble patterns) gives a better chance of detecting ATL activation, relative to a passive, resting baseline (Visser et al., 2010). Thus, we included control tasks in which participants processed scrambled stimuli. Finally, the dissimilarity between Experiment 1 and 2 (in tasks, stimuli, and nature of semantic congruity) was a key aspect of the experimental design. We leveraged the disparate contexts to test whether the ATL would be engaged by object recognition *generally* despite various surface dissimilarities. Within each experiment, we sought to identify the regions that *specifically* discerned semantic congruity. These examinations at the general *vs.* specific level was attained via contrasting ‘object tasks *vs.* scramble patterns’ and ‘congruent *vs.* incongruent’, respectively. This approach is akin to prior studies wherein researchers exploited stimuli differing in formats or modalities (words, objects, speech, and ambient sounds; e.g., Visser et al., 2012; Visser and Lambon Ralph, 2011) or tasks differing in requirements (social *vs.* non-social; see Rice et al., 2018) to tell apart the regions that respond invariantly *vs.* those that respond in a modality- or requirement-related way.

**Fig. 1:**
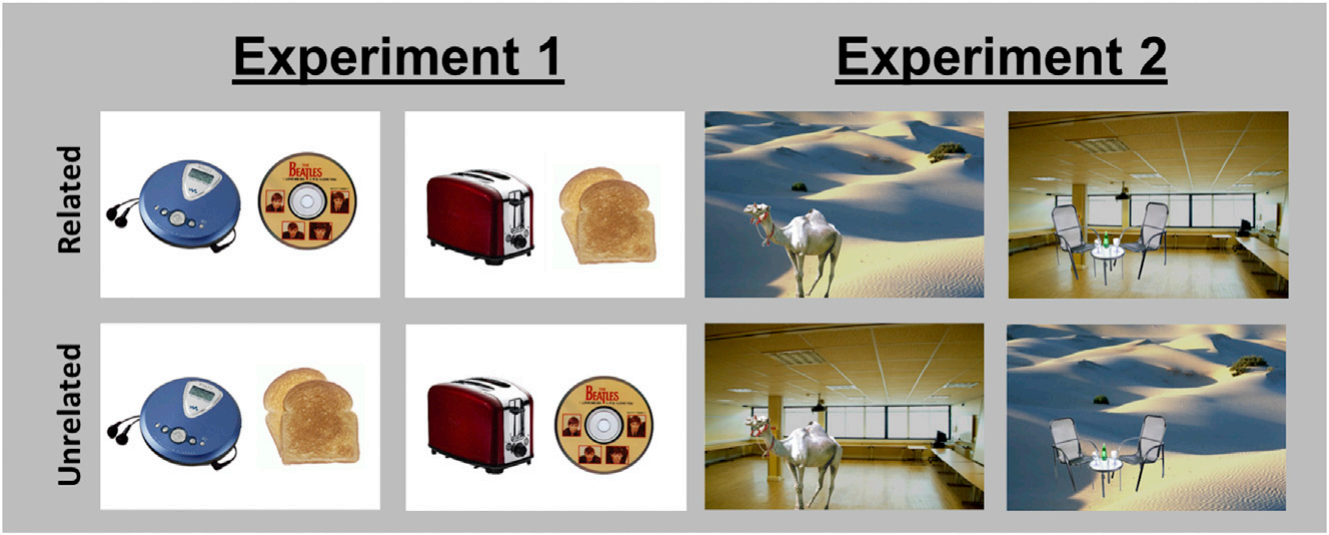
Example stimuli of Experiment 1 and 2. In Experiment 1, participants recognised a pair of visual items and selected the one that is an electronic device. The target (electrical) and foil (non-electrical) could be related or unrelated. Note that the target was equally likely to appear on the left or right. In Experiment 2, participants recognised the foreground item and indicated whether it is living or non-living. The foreground and background could be related or unrelated.

In Experiment 1, participants viewed a pair of visual stimuli in every trial, situated 4.2° to the left/right of the central cross. In the object recognition condition (2/3 of total trials, Fig. 1), the dual-object array consisted of two artefacts, one being an electronic appliance (target) and the other being a non-electrical item (foil), each subtended comparable size of 3.5°. The task was to identify which item of the pair was an electronic device by pressing one of the two designated buttons, with each key being equally likely to be used to indicate the target. We arranged the coupling between the targets and foils based on their relationship so that, in 50% of the object trials, they formed a semantically congruent pair that can be used together to perform some contextually appropriate actions (e.g., an iron and a shirt); in the other 50%, targets and foils were unrelated and could not be used together meaningfully (e.g., an iron and a rolling pin). Note that the task-relevant dimension (i.e., electrical *vs.* non-electrical) was orthogonal to the manipulation of semantic congruity, and the target location varied randomly and unpredictably on a trial-by-trial basis. In the non-object control task (the remaining 1/3 of trials), subjects viewed a pair of scrambled patterns created via breaking images of the object recognition task into 360 randomly assembled fragments. The two scrambled patterns of each pair had identical visual configuration; however, we scaled their sizes so that one was always 2% bigger than the other. The control task required participants to judge which stimulus looked bigger.

Stimuli were presented in a block design, controlled using E-Prime (Psychology Software Tools). A run of scanning consisted of 18 blocks of 18 s (related objects/unrelated objects/scrambled stimuli, each had 6 blocks), as well as six 12-sec resting periods, giving 396 s in duration. The order in which task-conditions and stimuli-sets were presented was counterbalanced across participants so that each task-condition was equally likely to appear in every possible position of the sequences, with images randomly drawn from a designated stimuli-set for a given scan and shuffled across blocks. Each block contained six trials. Each trial began with a fixation dot (0.5 s). Subsequently, a pair of stimuli (object images or scrambled patterns) was displayed for 2.5 s during which participants indicated the target (the electronic item or the visually bigger stimulus) by pressing a button on a MR-compatible response pad. All visual stimuli were displayed on a white background, presented via a mirror mounted above the head coil and projected through a screen at the foot of the scanner bed.

In a pilot behavioural study, we tested a separate group of seven volunteers (none participated in the fMRI study) using an extended set of object images. The participants viewed a pair of objects in each trial (one electrical, the other not) and rated the likelihood to which that the two artefacts could be employed together to perform certain meaningful actions, using a 5-point scale (1: least likely, 5: most likely). Only pairs that were found to be consistently rated as permitting meaningful actions (a rating of 4 or 5 across all 7 volunteers) were selected for the later fMRI experiments. This procedure yielded 216 pairs of objects for the final stimuli sets, consisting of 108 related and 108 unrelated pairs. Each individual object, be it electrical or not, appeared twice in the stimuli-sets, once presented with its semantically-related counterpart and the other time with an unrelated item. In the final stimuli-set, the average rating for related and unrelated objects was 4.37 (SE = 0.13) and 1.24 (SE = 0.03), respectively, with a significant difference in by-subject analysis (*t*_(6)_ = 22.69, *p* < 0.001). This difference was also manifest in by-item analysis for every individual participant (all *p*s < 10^−10^), supporting its robustness that related objects are reliably deemed as permitting meaningful actions.

In Experiment 2, participants viewed a photo (778 × 518 pixels, presented centrally) in each trial of the object recognition task (2/3 of total trials, Fig. 1). Each photo was comprised of a foreground item (either an animal or an artefact) embedded in a scene (either natural or artificial environment). All animals were wildlife species (elephant, lion, snake, etc.), and artefacts were pieces of household/office furniture. Natural scenes were seascapes or landscapes (beach, canyon, iceberg, etc.), and artificial scenes were various indoor environment (corridor, bathroom, garage, etc.). Participants identified whether the foreground item was living or non-living by pressing one of two designated buttons. We manipulated the combination between foreground items and background contexts so that, in 50% of the object trials, they formed congruent object-background associations (e.g., a deer on a meadow or a refrigerator in a kitchen); in the other 50%, they formed the opposite combination so that the foreground and background were semantically incongruent (e.g., a refrigerator on a meadow or a deer in a kitchen). Note that the task-relevant dimension (living *vs.* non-living) was orthogonal to semantic congruency and varied unpredictably between trials. In the non-object control task (1/3 of trials), we displayed scrambled patterns made by breaking images of the object recognition task into 640 randomly distributed fragments. The scrambled pattern (778 × 518 in size) was surrounded by a black frame, either 1 mm or 2 mm in thickness. For the control task, participants judged whether the frame was the relatively thinner or thicker one.

We employed the same stimuli as those used by Fabre-Thorpe et al. (Joubert et al., 2008; Rémy et al., 2013; Rémy et al., 2014). In these original studies, great care was taken to rigorously control for various low-level visual properties and to ascertain that they were equalised across conditions, including contrast, luminance, visual complexity, as well as the size and location of foreground item. These identical stimuli have been used to demonstrate robust congruency effects, replicated across multiple studies. Furthermore, we took care to select images so that there was no inappropriate image in the congruent condition (e.g., a crocodile on an iceberg). In the final stimuli-set for the present study, we used 216 images consisting of 108 related stimuli and 108 unrelated stimuli. Each foreground item and each background context appeared twice in the stimuli-set, once paired with its corresponding congruent counterpart and the other time paired with incongruent stimuli. Experiment 2 had the identical timing structure to Experiment 1 – in each run of scanning, stimuli of different task-conditions were presented in separate blocks of 18 s (6 trials; each trial consisted of 0.5-sec fixation and 2.5-sec target stimulus); each scan contained six 12-sec resting periods and 18 active-task blocks (6 related, 6 unrelated, and 6 scrambled). Similar to Experiment 1, we counterbalanced the order of all task-conditions and stimuli-sets across participants so that each condition and stimuli-set was equally probable to appear in every possible slot of the sequences; within each run, stimuli were randomly picked from an assigned image-set for a given run.

### 2.3 MRI acquisition

All scans were acquired using a 3T Phillips Achieva scanner equipped with a 32-channel head coil and a SENSE factor of 2.5. A dual-echo EPI sequence was used to maximise signal-to-noise ratio in the ventral ATL (Halai et al., 2014). Using this technique, each scan consisted of two images acquired simultaneously with different echo times: a short echo optimised to obtain maximum signal from the ventral ATL and a long echo optimised for whole-brain coverage. The sequence included 31 slices covering the whole brain with repetition time (TR) = 2.8 s, short/long echo times (TE) = 12/35 ms, flip angle = 85°, field of view (FOV) = 240 × 240 mm, resolution matrix = 80 × 80, slice thickness = 4 mm, and voxel dimension = 3 × 3 mm on the *x*- and *y*-axis. To reduce ghosting artefacts in the temporal lobe, all functional scans were acquired using a tilted angle, upward 45° off the AC-PC line. Functional scans of the two main experiments were collected over six runs; each run was 396-sec long during which 142 dynamic scans were acquired (alongside 2 dummy scans, discarded). To tackle field-inhomogeneity, a B_0_ field-map was acquired using identical parameters to the functional scans except for the following: TR = 599 ms, short/long TEs = 5.19/6.65 ms. Total B_0_ scan time was 1.6 min. A high-resolution T_1_-weighted structural scan was acquired for spatial normalisation, including 260 slices covering the whole brain with TR = 8.4 ms, TE = 3.9 ms, flip angle = 8°, FOV = 240 × 191 mm, resolution matrix = 256 × 163, and voxel size = 0.9 × 1.7 × 0.9 mm. Total structural scan time took 8.19 min.

### 2.4 Pre-processing

Analysis was carried out using SPM8 (Wellcome Department of Imaging Neuroscience). The functional images from the short and long echoes were integrated using a customised procedure of linear summation (Halai et al., 2014; Poser et al., 2006). The combined images were realigned using rigid body transformation (correction for motion-induced artefacts) and un-warped using B_0_ field-map (correction for field-inhomogeneity). The averaged functional images were then co-registered to each participant's T_1_ anatomical scan. Spatial normalisation into the MNI standardised space was achieved using the DARTEL Toolbox of SPM (Ashburner, 2007), which has been shown to produce highly accurate inter-subject alignment (Klein et al., 2009). Specifically, the T_1_-weighted image of each subject was partitioned into grey-matter, white-matter, and CSF tissues using SPM8's ‘Segmentation’ function; afterwards, the DARTEL toolbox was used to create a group template derived from all participants. The grey-matter component of this template was registered into the SPM grey-matter probability map (in the standard MNI stereotactic space) using affine transformation. In the process of creating the group's template brain using individual T_1_, for each individual DARTEL estimated ‘flow fields’ that contained the parameters for contorting native T_1_-weighted images to the group template. SPM8 deformation utility was then applied to combine group-to-MNI affine parameters with each participant's ‘flow fields’ to enable warping into the MNI space for each individual. The functional images were then resampled to a 3 × 3 × 3 mm voxel size. Smoothing was subsequently applied using an 8-mm Gaussian FWHM kernel, consistent with prior studies (e.g., Halai et al., 2014; Jackson et al., 2015).

### 2.5 GLM analysis

For each participant, contrasts of interest were estimated using general linear model (GLM) convolving a box-car function of all experimental conditions with a canonical haemodynamic response function, with resting periods modelled implicitly. Motion parameters were entered into the model as nuisance covariates. In addition, we added each participant's reaction time (RT) of all active-task performance as parametric modulators, allowing us to rule out any brain activation driven by task difficulty or cognitive effort when assessing the effects of experimental manipulation. Low-frequency drifts were removed using a high-pass filter of 128 s. Contrast images from the individual-level analyses were then submitted to random effect models in the group-level analyses.

### 2.6 Psychophysiological interaction

The PPI analysis was conducted to investigate how semantic congruity affected the connectivity between hub and spokes. In the GLM results, we found that the object recognition tasks robustly activated the anterior section of left fusiform gyrus (FG), reliably observed in both Experiment 1 and 2, and the locus of peak activity fitted nicely with prior findings of the typical location of ATL semantic hub (see the Results section for details). For PPI, we used the peak coordinates identified by the GLM analysis, and set the ‘seed’ of connectivity at the left anterior FG; we extracted its time-series of neural activity via deconvolving its BOLD signal and deriving the first eigenvariate. For each participant, we identified the local maxima of ATL activity, guided using the peak coordinates of group responses (Experiment 1: [−30, −9, −36]; Experiment 2: [−36, −15, −33]; both in the MNI space; for details see Section 3.2.1), and extracted the eigenvariate from a spherical ROI (radius = 6 mm) centred at the maximally responsive site. This procedure gave us the physiological factor (the eigenvariate/neural activity); we then convolved it with the psychological factor (contrast of task conditions: related context *vs.* unrelated context). This multiplication generated the interaction term – the psychophysiological (PPI) factor that accounted for task-driven changes in connectivity. This factor was used to identify possible ‘spoke’ zones whose neural connectivity with the ATL hub was modulated by semantic congruity. These three factors were used to construct a GLM model.

In addition to the three principal factors, we included additional regressors into the PPI analysis: (*i*) Six motion parameters that accounted for head movement. (*ii*) More importantly, the sizes of each participant's behavioural congruency effects were included as covariates to account for connectivity strength in relation to behavioural performance. In Experiment 1, semantic congruity primarily modulated accuracy; the covariate was hence defined as Accuracy_[related, %]_ – Accuracy_[unrelated, %]_. In Experiment 2, semantics primarily modulated RT; the covariate was thus defined as RT_[unrelated, ms]_ – RT_[related, ms]_. Note that, in both experiments, the covariate indices reflected the size of congruity effect, with a large numerical value of difference indicating a bigger effect, rather than the inversely-correlated measures of raw RT and accuracy. A whole-brain interrogation was performed to identify voxels whose activity could be explained by the PPI-interaction factor, without contamination of task condition and seed activity.

To evaluate whether the connectivity network detected by PPI fits with prior literature, we conducted analyses of region-of-interest (sphere, radius = 6 mm) based on the coordinates from representative studies about action and place processing, examining whether the PPI estimates were evident at those literature-defined ROIs. For Experiment 1, many participants reported in the post-scanning debriefing conversation that the display of congruent objects prompted them to engage in motor imagery of performing customary actions to use the two items together (e.g., putting a disk into a CD player by hand) whereas incongruent objects elicited little imagery. We speculated that such motor imagery might reinforce connectivity between the ATL hub and motor regions. Thus, we selected three sets of coordinates based on the meta-analysis by Hétu et al. (2013) on motor imagery, peaking at the left inferior frontal gyrus [−52, 8, 12], the left precentral gyrus [−50, −2, 42], and the right precentral gyrus [56, 2, 44] – all belong to the broad action-semantics system. For Experiment 2, we speculated that the ATL hub would be linked with regions implicated in processing the mnemonic aspects of a place, such as the retrosplenial cortex that is known to represent one's familiarity with a place (for review, see Epstein, 2014). Thus, we selected the coordinates based on two recent fMRI studies that are focused on the function of retrosplenial cortex in place-related memory: [−8, −48, 4] from Shine et al. (2016) and [−6, −56, 18] from Chrastil et al. (2015).

### 2.7 Dynamic causal modelling

In the PPI analysis, we discovered that the ATL hub became more tightly connected with the action- and place-related spoke regions when the contexts encouraged the retrieval of action and place knowledge, respectively. Based on the results of PPI, we next used DCM to investigate causative interactions between the hub and spokes. Analyses were performed using DCM10, incorporated in SPM8. The first stage of analysis involved extracting the time-course of activation of the hub and spokes for each participant. This process was guided using the peak activation sites found in the initial GLM results and PPI analyses. Specifically, for each individual and each experiment we extracted the first eigenvariate (deconvolved neural activity, using the default algorithms of SPM8) from a spherical ROI (radius = 6 mm) centred at the maximally responsive point of the ATL (identified by the GLM contrast) and action- and place-related spokes (identified by the PPI analyses). Constrained based on the local maxima of PPI connectivity, for Experiment 1 the spoke node was located at the cluster peak in the right precentral gyrus (a region crucial for pre-motor planning and motor imagery; see Ishibashi et al., 2016), whereas for Experiment 2 the spoke was located at the retrosplenial cortex (an area crucial for remembering a place and its associated elements, see Epstein, 2014). For each experiment, the DCM contained only two nodes – the ATL hub and the functionally specific spoke. The nodes of hub and spoke were set to be mutually connected, allowing us to test whether causative impact existed in only one or both directions. In addition, there is ample evidence that incoming visual signals are routed to the ATL through the big fibre bundle of inferior longitudinal fasciculus (IFL), linking the ATL with the occipitotemporal visual system, and that the integrity this input-pathway correlates with semantic performance (e.g., Almairac et al., 2015; Bajada et al., 2015; Herbet et al., 2018; Hodgetts et al., 2017; Kravitz et al., 2013). Therefore, based on the plenitude of evidence about the ILF directly and swiftly feeding forward to the ATL, we set the ATL as the entry of triggering input, in accordance with the anatomical infrastructure of the human brain.

The straightforward, two-node DCM structure, plus the empirical evidence that demonstrates the existence of PPI connectivity between the two nodes, provided an appropriate context for focusing directly on the DCM parameters, following the recommendation of DCM guidelines (Stephan et al., 2010). Here we focused on two sets of parameters that the DCM yielded: (*i*) the *intrinsic* connectivity, representing the inherent coupling strength between the hub and spoke that is devoid of the perturbation of semantic contexts; (*ii*) the *modulatory* connectivity, representing an alteration to connectivity strength driven by the contextual difference of congruent *vs*. incongruent that is imposed upon the intrinsic linkages between network nodes.

## 3 Results

### 3.1 Behavioural results

In Experiment 1, semantic congruency concerned the relationship between objects and actions. While congruency did not modulate RT (*t*_(19)_ = −1.24, *p* = 0.23; Cohen's *d* = 0.28; related: 759 ms, unrelated: 776 ms), it drove a significant effect in accuracy (*t*_(19)_ = 3.94, *p* = 0.001, *d* = 0.88), with object identification being more accurate for related objects that allowed meaningful actions (96%) than unrelated pairs (93%).^1^ In Experiment 2, semantic congruency concerned the relationship between objects and places. In RT, we found a significant effect (*t*_(19)_ = −4.38, *p* < 0.001, *d* = 0.98) – identifying objects in semantically congruent scenes (671 ms) was faster than in incongruent scenes (698 ms). In accuracy, we found a significant but much more subtle effect that identification was more accurate in the congruent (96%) than incongruent condition (94%; *t*_(19)_ = 2.34, *p* = 0.03, *d* = 0.52). It is important to note that such congruency effects have been replicated numerous times in previous studies that intermingled congruent and incongruent trials (e.g., Davenport, 2007; Davenport and Potter, 2004; Joubert et al., 2008; Rémy et al., 2013; Rémy et al., 2014). Thus, while we opted to use a block design to optimise detection of functional connectivity, we were still able to replicate the congruency effects. This suggests that the effects are highly robust that they do not depend on a specific mode of presentation, be it blocked or intermingled. Moreover, previous studies that used brief presentation (e.g., 100 ms in Rémy et al., 2014) are open to the criticism that congruency effects were driven by guesswork (e.g., due to brief duration that hinders object recognition, participants simply use background contour as a clue to infer possible items, such as an urban-looking background is more likely to contain artefacts than animals, while object recognition *per se* is unaffected by background). With a longer 2.5-sec display that allowed sufficient visual information to accrue, in the present study we ensured that the robust effects cannot be due to guesswork at a decisional level. Also note that in all of our fMRI analyses, RTs (including performance on the control tasks) were modelled as parametric modulators, which controlled for variation of brain activity associated with response latency.

### 3.2 fMRI analysis

#### 3.2.1 Object recognition engages the ATL semantic hub

First and foremost, we verified whether the object recognition tasks engaged the ATL semantic hub by contrasting the brain's overall response to the object recognition tasks (combining congruous and incongruous trials) against the control scrambled-pattern tasks. Statistics was thresholded at *p* < 0.05 (FWE-corrected for multiple comparisons) at the cluster level and *p* < 0.001 at the voxel level. We detected, in both experiments, robust activity of the ATL hub driven by object recognition.

**Fig. 2:**
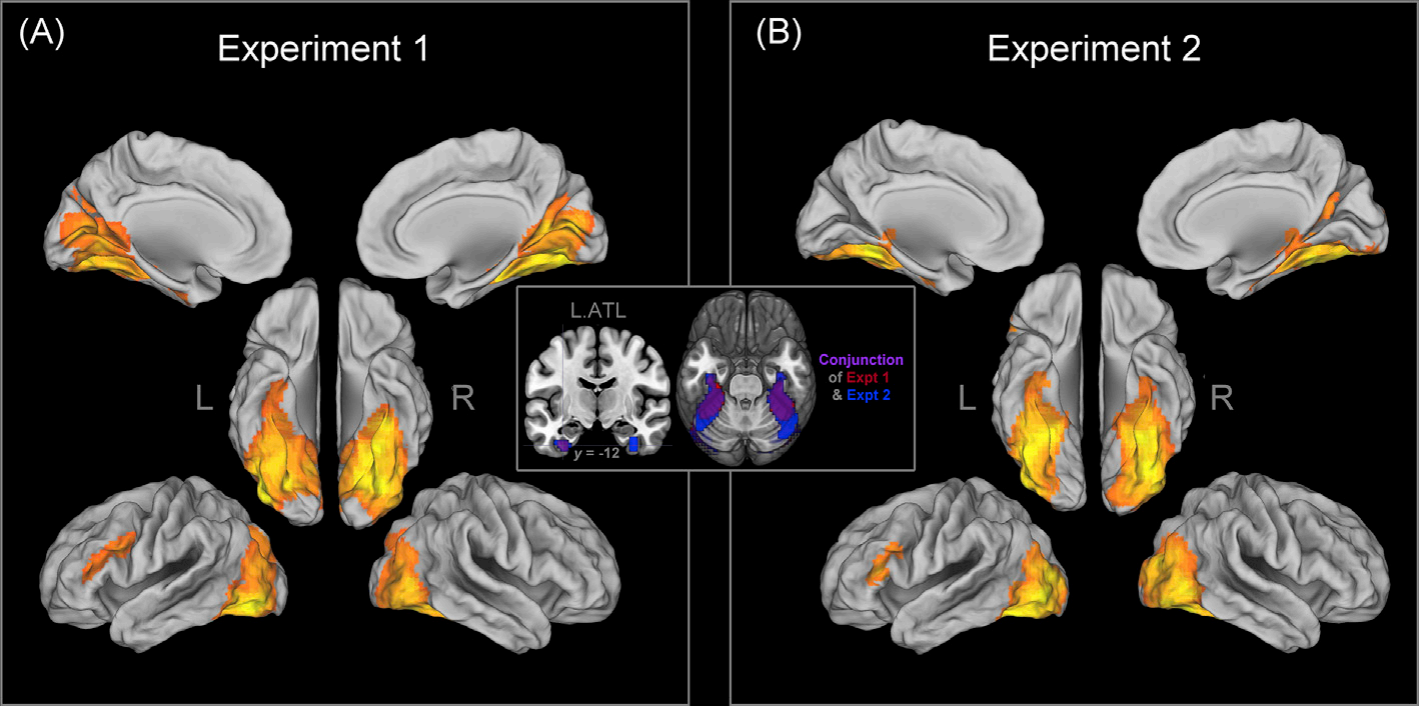
Whole-brain analysis identified regions activated by object recognition, contrasting against scrambled-pattern control tasks (**A**: Expriment 1; **B**: Experiment 2). The thresholds were set at *p* < 0.001 at the voxel level and *p* < 0.05 (FWE-corrected for multiple comparisons) at the cluster level. The middle inset-box reveals that a shared ‘hub’ region at the left ATL was commonly engaged by the two experiments (the purple-shaded area).

Specifically, for Experiment 1, object recognition elicited expansive swathes of activity extending across the ventral occipitotemporal cortices, merging as a single extensive cluster that encompassed much of the bilateral visual cortex (cluster size/*k* = 145,206 mm^3^). As shown in Fig. 2A, this contrast revealed the classic regions of object perception (the LOC and posterior/middle FG), spreading medial-ventrally to the lingual gyrus and cuneus, also dorsal-laterally to the transverse occipital sulcus. Critically, in the left hemisphere, this significant cluster unfolded from these posterior visual cortices, stretched along the medial-parahippocampal flank of ventral cortical pathway, and continued into the territory of the ATL hub (the anterior FG and the perirhinal cortex). The local maxima of this left ATL activity peaked at *T* = 5.53, situated at [−30, −9, −36]. A strikingly similar pattern was observed in Experiment 2 (Fig. 2B) – the ‘objects *vs.* control’ contrast revealed significant activity along the bilateral ventral pathway (*k* = 51,749 mm^3^ in the left hemisphere, and 61,749 mm^3^ in the right hemisphere), covering the posterior object-sensitive regions and the anterior semantics-sensitive regions. Particularly, in the left hemisphere, we observed robust activation of the ATL, peaking at the anterior FG and nearby perirhinal region, with local maxima (*T* = 5.27) detected at [−36, −15, −33]. This task also engaged the right ATL homologue in Experiment 2, peaking (*T* = 5.12) at [36, −15, −30], although right ATL activation did not survive the applied threshold in Experiment 1. To highlight the overlap between the ATL clusters of both experiments, we display a conjunctive map in the middle inset-box of Fig. 2. It is noticeable that the left ATL, particularly its rostral FG sector, was reliably recruited by object recognition in both experiments (see the purple-shaded area that indicates the locus of overlap in ATL activities). The locale of this overlap also concurs with prior literature regarding the semantic hub in healthy and diseased brain (e.g., Binney et al., 2016; Chiou et al., 2018; Mion et al., 2010; Rice et al., 2018; Visser and Lambon Ralph, 2011).

Taken together, we found that object recognition not only recruited the occipitotemporal regions specialised for visual configuration processing (Grill-Spector and Weiner, 2014; Kravitz et al., 2013) but also the ATL known for its role in semantic cognition (Lambon Ralph et al., 2017). It is striking that, while the task demands and stimuli configurations widely differed between Experiment 1 and 2, an extensively overlapped network participated in both object recognition tasks. In particular, using the dual gradient-echo protocol that surmounted signal loss, we detected robust activation of the ATL that has long been overlooked in the literature. Reliable ATL activity, especially the left ATL sector that was replicated across experiments, underscores its ubiquitous engagement in a wide range of contexts involving object recognition, irrespective of variation in tasks and stimuli.

#### 3.2.2 Semantic congruity engages the action- and place-related spokes

Next, we identified areas sensitive to semantic congruency by contrasting related *vs*. unrelated trials, thresholded using *p* < 0.005 for voxel intensity and corrected for cluster-level multiple comparisons at *p* < 0.05. Relative to the more stringent threshold that we used to detect the brain's all-inclusive general response to object recognition (i.e., comparing the aggregate of congruent and incongruent trials against the control tasks of scrambled patterns), this relatively lenient threshold was applied to compare the congruent *vs.* incongruent trials that had nearly identical perceptual configurations and only differed in the subtle semantic aspect. We sought to identify the brain area that discerns such semantic granularity.

**Fig. 3:**
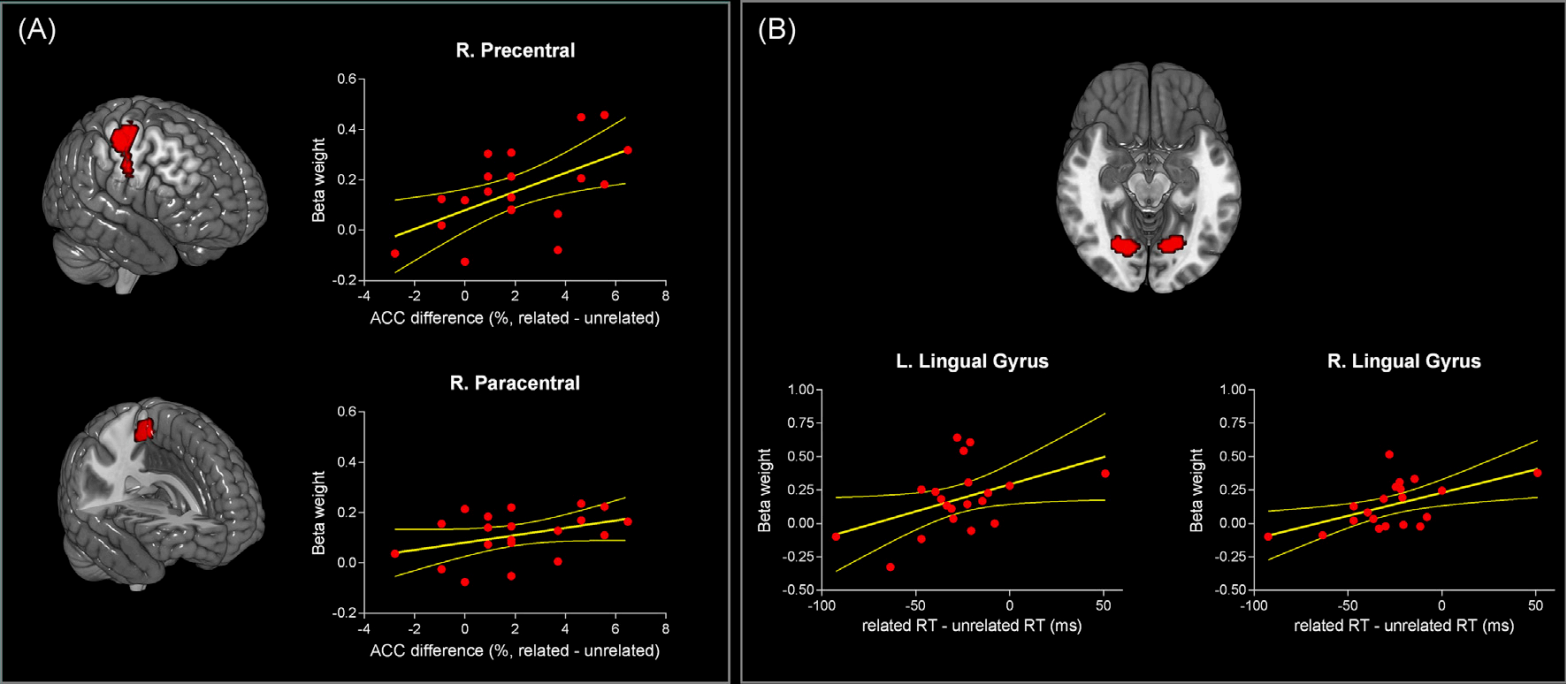
**(A)** The rendered images display the results of whole-brain search identifying regions more active for related, action-permitting object pairs, compared against unrelated objects. The scatter plots display the correlational relationships between *β* responses (extracted from the two active clusters of the right motor cortex) and the size of behavioural effect. **(B)** The rendered images display the results of whole-brain search identifying regions more active for related, object-scene composites, compared against unrelated stimuli. The scatter plots display the correlational relationships between *β* responses (extracted from the bilateral LG) and the size of behavioural effect. The thresholds were set at *p* < 0.005 at the voxel level and further constrained for cluster-level multiple-comparisons at *p* < 0.05.

In Experiment 1, we found object pairs that permitted meaningful actions elicited greater activity in two action-related regions of the right hemisphere, relative to unrelated object pairs. Both clusters belong to the broad frontoparietal action-related network – one active cluster was located in the precentral lobule (cluster peak: [42, −15, 57], *k* = 4455 mm^3^, Fig. 3A); the other active cluster was situated in the paracentral lobule (cluster peak: [9, −33, 57], *k* = 3213 mm^3^, Fig. 3A). Interestingly, we found that response amplitude (*β* estimate) within these motor clusters was correlated with the size of behavioural congruency effect, manifested as a statistically significant correlation at the peak of the precentral cluster (Pearson's *r* = 0.57, *p* = 0.008) and a weak trend at the paracentral cluster (Pearson's *r* = 0.39, *p* = 0.09). As Fig. 3A illustrates, participants who achieved higher accuracy for related stimuli tended to show greater activation in these two motor-system clusters.

In Experiment 2, we found that, compared to unrelated contexts, visual items embedded in semantically-related scenes elicited greater activation in the bilateral lingual gyri (LG), known to be parts of the ventromedial place-processing system (Epstein, 2014): the left LG cluster peak: [−15, −69, −3], *k* = 1593 mm^3^; the right LG cluster peak: [21, −69, −3], *k* = 1944 mm^3^ (Fig. 3B). Interestingly, we again observed a reliable relationship between brain and behaviour such that the beta strength of these lingual clusters correlated with the size of behavioural effect. Significant correlations were detected both at the peaks of the left LG (Pearson's *r* = 0.45, *p* = 0.04) and right LG (Pearson's *r* = 0.55, *p* = 0.01). As Fig. 3B illustrates, participants who spent longer responding to the related stimuli tended to show greater activation for these stimuli in the bilateral LG. This also concurs with the general observation that slower reaction latency is associated with greater BOLD signal of relevant regions (e.g., Yarkoni et al., 2009).

Together, these analyses revealed that the ATL hub was commonly engaged by both kinds of object recognition tasks, consistent with its domain-general nature. By comparison, regions known for their roles in action- and place-related processing are differentially engaged to tackle distinct types of semantic congruity, consistent with their domain-specific nature.

#### 3.2.3 PPI

Using the connectivity analysis of PPI, we sought to verify these two speculations: (*i*) whether the common hub liaises with different spoke modules under different semantic contexts via long-range connection, which would reveal distinct networks for action and place knowledge; (*ii*) whether the strength of functional connectivity with the ATL hub correlates with behavioural performance. The seeds of connectivity were set at the left ATL that showed robust activation to object recognition across experiments. Statistics were thresholded at *p* < 0.005 for voxel intensity and further constrained for cluster-level multiple comparisons at *p* < 0.05, as per our previous analysis that was focused on the comparison of congruent *vs.* incongruent condition (for the complete list of activated clusters, see Table 1, Table 2).

**Table 1:** Significantly active cluster identified by the PPI analysis of Experiment 1.

Location	Extent (mm^3^)	Z-value	MNI coordinates (x, y, z)		
**PPI effect: Related > Unrelated**					
L. Precentral gyrus	1620	4.11	−51	−9	45
R. Precentral gyrus	3402	3.88	54	−6	27
		3.24	63	−6	15
L. Precentral gyrus	2430	3.18	−54	−12	9
		3.08	−57	−3	18
**Parametric modulator: size of behavioural effect**					
L. Inferior frontal gyrus	4158	4.56	−27	24	−9
		3.73	−21	57	3
R. Precentral gyrus	2133	3.31	45	0	36
		3.23	54	0	30
R. Precentral gyrus	1809	3.30	21	−21	60
		3.28	12	−12	69
R. Superior parietal lobule	1890	3.22	24	−45	57
		3.19	9	−51	51
R. Supramarginal gyrus	1863	3.11	54	−30	42
		3.10	60	−24	33

**Table 2:** Significantly active cluster identified by the PPI analysis of Experiment 2.

Location	Extent (mm^3^)	Z-value	MNI coordinates (x, y, z)		
**PPI effect: Related > Unrelated**					
L. Precuneus	1701	4.40	−15	−72	33
L. & R. Precuneus	2646	3.21	18	−69	39
		3.20	−3	−51	48
**Parametric modulator: size of behavioural effect**					
L. Retrosplenial cortex	1728	5.23	−6	−54	12
		3.02	−3	−42	27

**Fig. 4:**
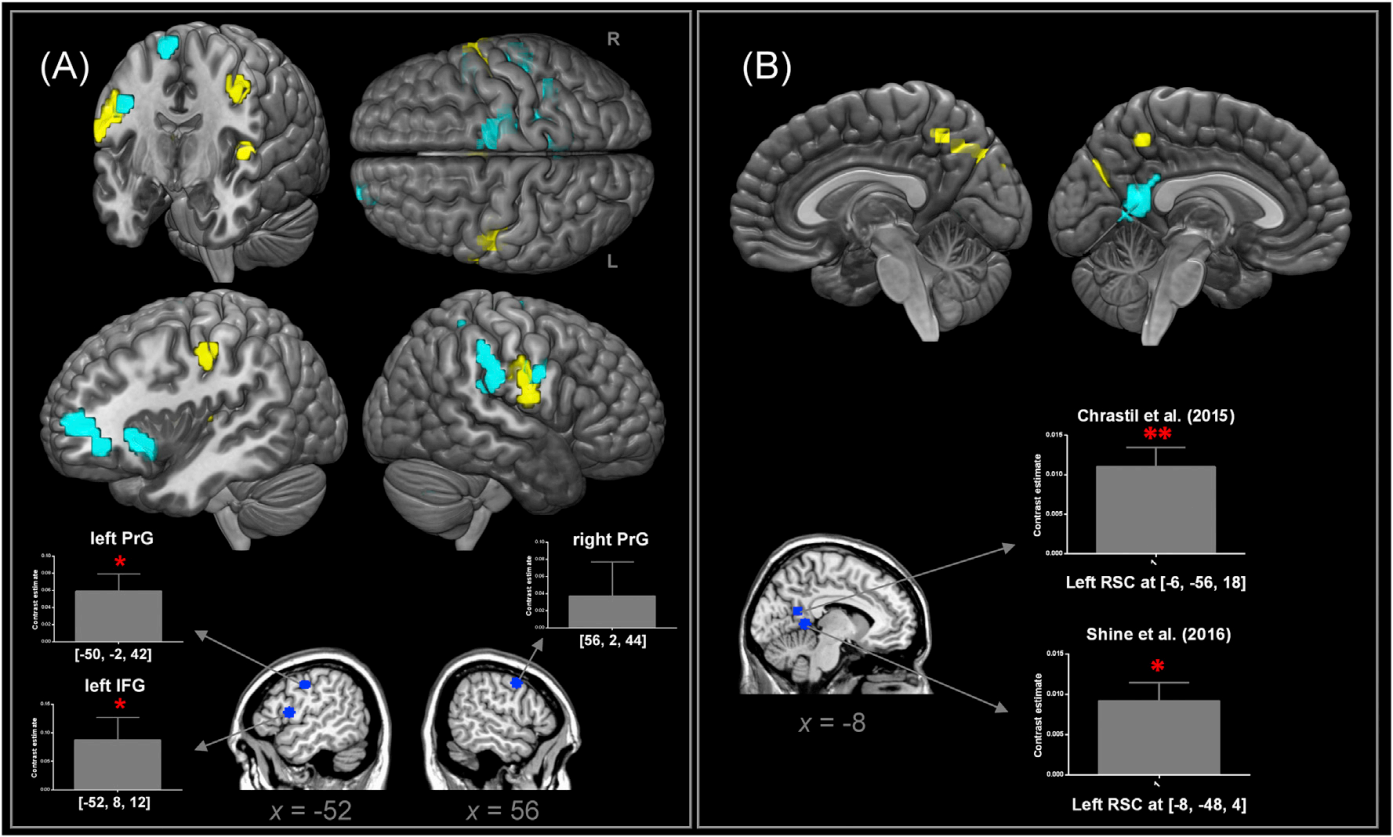
**(A)** The rendered images display the results of PPI analysis, identifying regions significantly more connected to the ventral ATL seed for related, action-permitting objects than for unrelated objects (yellow) and the regions significantly modulated by the behavioural congruency effect (cyan). Below the rendered images are the results of ROI analysis based on the meta-analysis of Hétu et al. (2013) on the motor system. **(B)** The rendered images display the results of PPI analysis, identifying regions significantly more connected to the ventral ATL seed for related, object-scene composites than for unrelated images (yellow) and the regions significantly modulated by the behavioural congruency effect (cyan). Below the rendered images are ROI analysis based on Shine et al. (2016) and Chrastil et al. (2015) concerning place-processing. The thresholds of PPI were set at *p* < 0.005 for voxel intensity, further constrained using *p* < 0.05 for cluster-level multiple comparisons.

In Experiment 1 (Fig. 4A), the PPI effect was manifested in regions of the frontoparietal action-related network – these clusters showed stronger connectivity to the ATL seed when the displayed objects were related and implied meaningful actions, compared to unrelated pairs. These regions included the bilateral precentral gyri and extended well into the postcentral gyri and the left parietal-operculum. Moreover, the covariate regressor of individual task performance (the size of behavioural congruency effect) significantly modulated the action-related network. Participants who showed a bigger congruency effect tended to have strengthened functional coupling between the ATL and various areas of the extended action system, including the inferior frontal gyrus (IFG; peaking at *pars orbitalis* and extending to the frontal pole) and the right parietal clusters (the superior parietal and supramarginal lobules). This pattern of connectivity suggests that, when stimuli connoted actions, the ATL became more tightly coupled with various frontoparietal regions, whose location dovetails nicely with the system of action-semantics (van Elk et al., 2014). Further, the coupling ramped up for individuals showing a bigger congruency effect. We also examined whether such PPI effect was manifest at the literature-defined ROIs relevant to action imagery (Hétu et al., 2013). Results (Fig. 4A) show significant effects at two ROIs: the left IFG (*t*_(19)_ = 2.13, *p* = 0.04) and at the left precentral gyrus (*t*_(19)_ = 2.73, *p* = 0.01).

In Experiment 2 (Fig. 4B), the PPI effect was revealed in the bilateral precuneus cortices that showed enhanced connectivity with the ATL when the foreground items fitted their scenes semantically. Crucially, task performance (the size of behavioural congruency effect) significantly affected the left retrosplenial cortex (RSC) – participants who showed a bigger behavioural congruency effect tended to have stronger coupling between the ATL and RSC. These data are in accordance with previous results implicating the medial sector of the brain (including both the RSC and precuneus) in representing visual scenes (e.g., Summerfield et al., 2010; Walther et al., 2009). Moreover, it revealed the ATL's pivotal status in mediating behaviour – the ATL reinforced its dialogue with the place-sensitive system, with the strength of neural connectivity mirrored in the magnitude of behavioural congruency effect. We also tested whether the PPI effect reached significance at the literature-defined ROIs based on two fMRI studies about place memory. Results (Fig. 4B) reveal significant effects at both of the locations identified by Shine et al. (2016; *t*_(19)_ = 2.14, *p* = 0.04) and Chrastil et al. (2015; *t*_(19)_ = 3.19, *p* = 0.005).

#### 3.2.4 DCM

Results of the PPI analysis detected that the ATL semantic hub was more intimately coupled with the frontoparietal action-related and medial place-related cortical zones when participants recognised objects in contexts implicating action and place knowledge, respectively. However, while PPI revealed the statistical dependency between the hub and spokes, the nature of such connectivity was still correlative (even though the PPI's regression model implied directional influences). To investigate the directional/causative impacts between the hub and spokes, we carried out DCM and focused on these questions: (*i*) whether the strength of intrinsic connectivity between hub and spokes is symmetrical or asymmetrical; (*ii*) whether the connectivity was modulated by semantic congruity; (*iii*) whether the fluctuation in neural connectivity was reflected in the size of behavioural congruity effect.

**Fig. 5:**
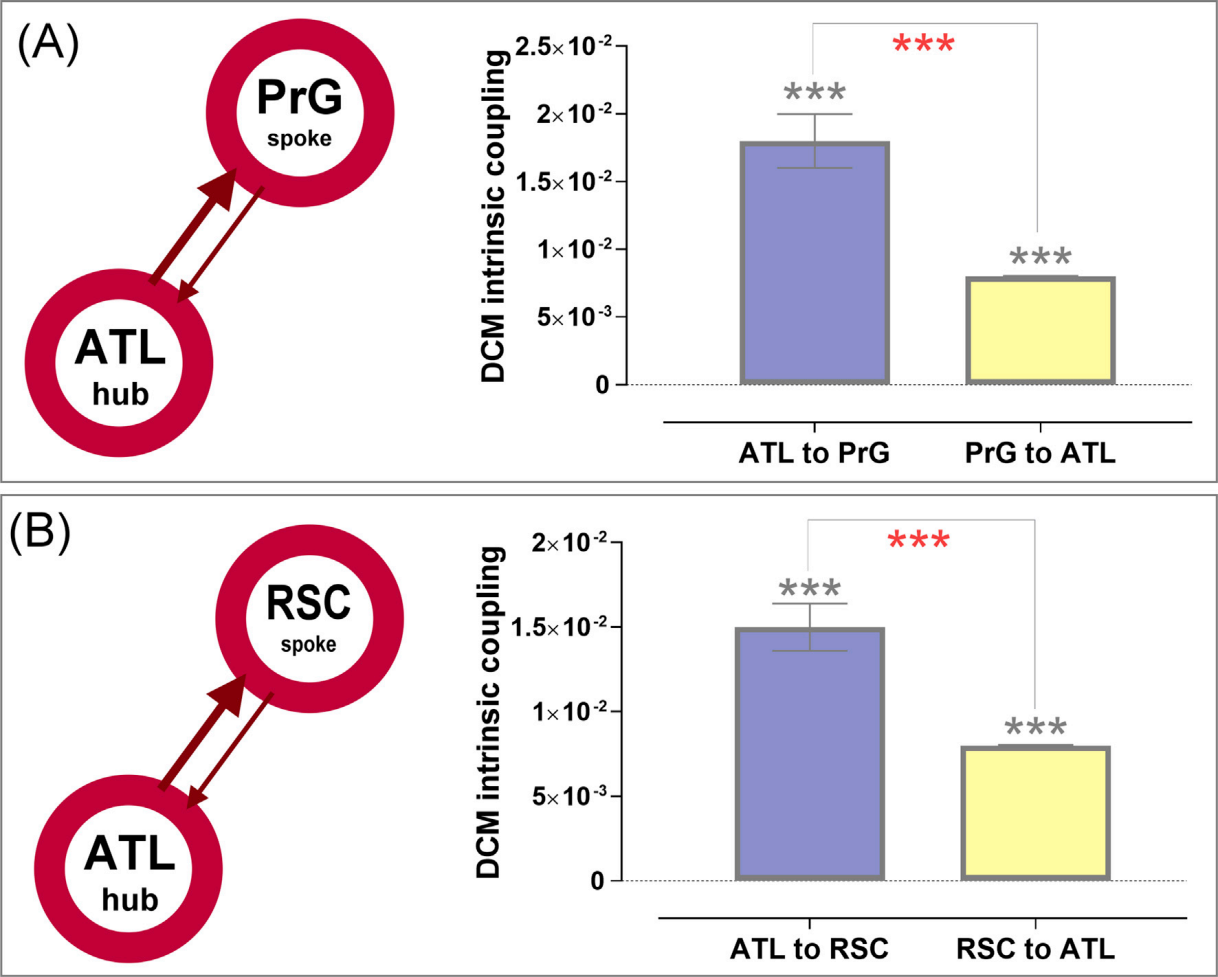
Analysis of DCM on the *intrinsic* coupling between the domain-general ATL hub and content-specific spoke (**A**: Experiment 1, with the action-related precentral gyrus / PrG being the spoke; **B**: Experiment 2, with the place-related retrosplenial cortex / RSC being the spoke). The bar-charts show the magnitude of intrinsic coupling parameters. The diagram illustrates the structure of DCM; the thickness of arrows in the diagram depicts the outcome that the ATL's directional effect on the spoke was significantly greater than the effect the other way around. Localisation of the hub and spoke's coordinates was guided based on the results of prior GLM and PPI analysis.

First, we focused on intrinsic connectivity (inherent linkage without contextual perturbation), checking whether causative linkage existed (i.e., significantly different from the null hypothesis) reciprocally or not, and whether there was any asymmetry in the possible mutual connection. As shown in both Fig. 5A and B, it is evident that, in both experiments, the influences between the ATL hub and spokes are mutual and excitatory (significantly greater than zero in both ways, all *p*s < 10^−8^), observed both from the hub to spoke, as well as from the spoke to hub. Interestingly, there was a clear asymmetry in the ‘loudness’ with which the hub and spokes speak to each other, consistently found in both Experiment 1 and 2. Specifically, in Experiment 1 the directional impact that the ATL hub exerted on the action-related PrG spoke was significantly greater than the opposite effect from the spoke to hub (*t*_(19)_ = 5.11, *p* < 0.0001). The asymmetry was also found in Experiment 2: the impact that the ATL hub wielded on the place-related RSC spoke was significantly greater than the inverse effect from spoke to hub (*t*_(19)_ = 5.31, *p* < 0.0001). Note that while the feedback signals from spoke to hub was comparatively weaker, in both experiments they were still highly significantly above zero.

**Fig. 6:**
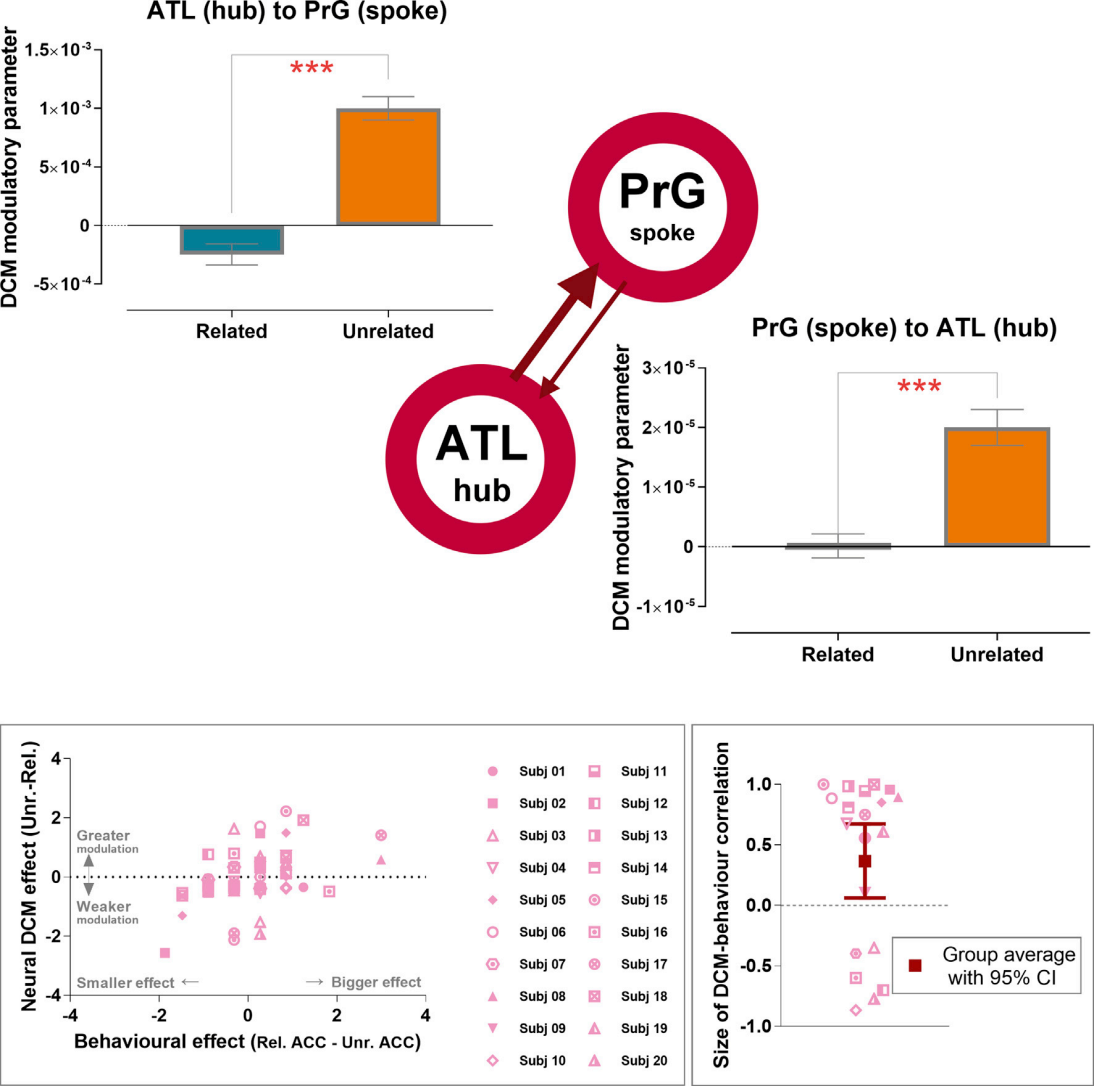
Experiment 1: Analysis of DCM on the *modulatory* coupling between the ATL hub and action-related spoke (the precentral gyrus). The two bar-charts show the magnitude of modulatory coupling parameters by directionality (hub-to-spoke, spoke-to-hub) and contexts (related, unrelated). The thickness of arrow in the diagram depicts the outcome that the hub-to-spoke connection was more modulated by contexts. Below the diagram: **(*****i*****)** The left inset-box describes the size of behavioural congruency effect (the difference in accuracy of related *vs.* unrelated context) in relation to the effect at neural level (the difference in DCM modulatory parameters, related *vs.* unrelated). This contains 60 data-points (20 participants × 3 runs of scanning). For illustrative purpose, the values are normalised, transformed to standard *Z*-scores. **(*****ii*****)** The right inset-box describes the values of DCM-behavioural correlation. This contains 20 data-points; each represents an individual's correlation across scanning runs. Note that the group's average is significantly above the chance level (no correlation).

**Fig. 7:**
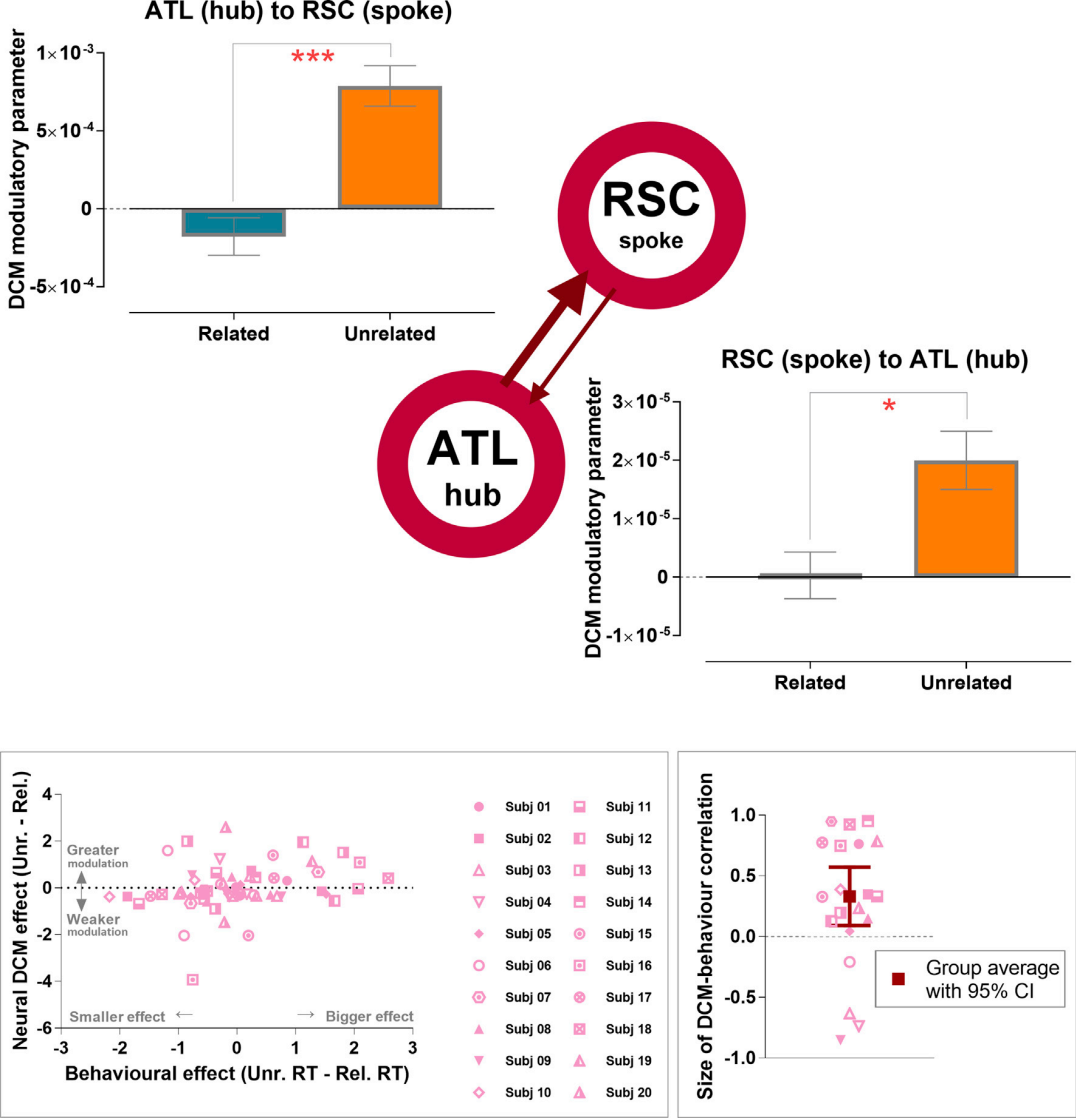
Experiment 2: Analysis of DCM on the modulatory coupling between the ATL hub and place-related spoke (the retrosplenial cortex). The two bar-charts show the magnitude of modulatory coupling parameters by directionality (hub-to-spoke, spoke-to-hub) and contexts (related, unrelated). The thickness of arrow in the diagram depicts the outcome that the hub-to-spoke connection was more modulated by contexts. Below the diagram: **(*****i*****)** The left inset-box describes the size of behavioural congruency effect (the difference in RT of related *vs.* unrelated context) in relation to the effect at neural level (the difference in DCM modulatory parameters, related *vs.* unrelated). This contains 60 data-points (20 participants × 3 runs of scanning). For illustrative purpose, the values are normalised, transformed to standard Z-scores. **(*****ii*****)** The right inset-box describes the values of DCM-behavioural correlation. This contains 20 data-points; each represents an individual's correlation across scanning runs. Note that the group's average is significantly above the chance level (no correlation).

Second, we examined the modulatory parameters (changes to connectivity strength evoked by experimental contexts), checking whether the reciprocal links are susceptible to the modulation of semantic contexts (related *vs.* unrelated), and whether their susceptibility differed between the paths of hub-to-spoke and spoke-to-hub. As shown in Fig. 6, in Experiment 1 we found that the modulatory parameters were exceedingly greater when the objects were not adequately associated (a main effect of unrelated > related: *F*_(1,19)_ = 40.09, *p* < 0.001), and that the hub-to-spoke path is more susceptible to contextual modulation than the spoke-to-hub path (*F*_(1,19)_ = 37.97, *p* < 0.001; note the different scales between the 2 bar-charts). Importantly, these two factors significantly interacted with each other (*F*_(1,19)_ = 40.04, *p* < 0.001), indicating the contextual effect (unrelated > related) being more potent for the hub-to-spoke route than the reverse. A strikingly consistent pattern was observed on the data of Experiment 2. As shown in Fig. 7, modulatory parameters were significantly greater for the hub-to-spoke route (*F*_(1,19)_ = 52.99, *p* < 0.001, as evident in the different scales of the bar-charts) and when the stimuli showed discordance between objects and backgrounds (*F*_(1,19)_ = 15.50, *p* = 0.001). In keeping with the asymmetry found in Experiment 1, there was a significant interaction that incongruity (unrelated > related) had a more powerful impact on the hub-to-spoke route than the opposite route (*F*_(1,19)_ = 16.27, *p* < 0.001).

Third, we scrutinised whether the DCM modulatory parameters systematically co-varied with the behavioural congruency effects. In the preceding analyses, we learnt that the ATL hub amplified its regulation on the spokes when the stimuli conveyed semantically incongruous message (e.g., a blender paired with a leash, or a cradle in a jungle). We speculated that a heightened level of modulation at the neural level (DCM modulatory parameters, hub-to-spoke: unrelated > related) might systematically vary with behavioural congruency effects. Thus, for each participant and each run of scanning, we calculated the size of the behavioural effect of that given run, and then correlated behavioural outcome with the run-specific DCM modulatory parameter. This correlational analysis enabled us to verify whether, within individual performance, fluctuation in the size of behavioural effect (across the three runs of scanning) was mirrored in the neural connectivity gauged by DCM. In Experiment 1, the systematic co-variation was reliable – when participants made more error to unrelated stimuli (hence, a bigger congruency effect of behaviour), the DCM modulatory parameters tended to become greater under such unrelated contexts (see the left inset-box of Fig. 6). Moreover, while the magnitude of correlation between behavioural effects and DCM parameters varied somewhat amongst individuals, a test at the group-level showed that this trend was statistically reliable (*t*_(19)_ = 2.35, *p* = 0.03, see the right inset-box of Fig. 6). A consistent pattern was replicated in the data of Experiment 2: when participants required longer latency to react to unrelated stimuli (a bigger congruency effect), the DCM modulatory parameters also escalated under the unrelated contexts (the left inset-box of Fig. 7). A group-level test confirmed its reliability across participants (*t*_(19)_ = 2.27, *p* = 0.03, see the right inset-box of Fig. 7). We elaborate on the interpretation regarding these brain-behaviour correlations in the Discussion section.

## 4 Discussion

There is abundant evidence showing that semantic knowledge is underpinned by two major pillars (see Lambon Ralph et al., 2017, for comprehensive review): (*i*) A constellation of modality-specific and functionally-specialised regions that constitute the spokes; and (*ii*) the ventral section of the ATL, primarily the realm of anterior FG and perirhinal cortex, that functions as a transmodal hub (for an alternative construal of hub-and-spoke interactivity using a multi-level framework, see Reilly et al., 2016b; also see Kiefer and Pulvermüller (2012) for discussion about the ATL's representational nature). While much support from patient research (Guo et al., 2013; Mion et al., 2010) and brain stimulation (Chiou and Lambon Ralph, 2016; Pobric et al., 2010) has confirmed the necessity of a bipartite division of labour, little is understood about how the hub and spoke interact with one another. In the present study, we investigated the neuro-dynamics between the hub and spokes when participants processed action and place knowledge in two fMRI experiments. We found that, despite task demands and stimuli configurations widely differing between the two experiments, a common hub situated in the left rostral-FG was recruited, which coincided closely with the locus of the ATL hub documented in prior neuropsychology and neuroimaging literature. Critically, while the left ATL was commonly engaged in both tasks, its functional connectivity with the spokes varied with different types of knowledge. The ATL amplified its connectivity with the frontoparietal action-sensitive regions in the context of action knowledge, whereas it became connected with the medial place-sensitive regions for the retrieval of place knowledge. Further, an asymmetry was consistently found in their interaction that the hub's causative impacts on spokes dwarfed the opposite impacts, suggesting the hub's dominant status in the dynamics. These results also dovetail nicely with previous multivoxel decoding results that the ATL hoards information about objects' typical location and customary action (Peelen and Caramazza, 2012), and that the ATL serves as a switchboard to conjoin information from remote sources (Wang et al., 2017). At both levels of between-individuals (variation amongst participants) and within-individual (variation across the runs of scanning, specific to each participant) we found that the strength of functional coupling between the hub and spoke correlated with the size of behavioural congruency effect. Taken together, our findings revealed the fluidity of the ATL hub in adjusting its functional connectivity depending on different contents of semantic knowledge. The neuro-dynamics between the ATL hub and content-specific spokes reliably correlated with behavioural congruency effects, providing further support for the functional relevance to human behaviour that the hub-and-spoke system has.

Our findings provide further empirical evidence showing the mechanistic operation of the hub and spoke system of the human brain. In both experiments, the nuanced difference of semantic congruity (related *vs.* unrelated contexts) could only be manifested in the domain-specific ‘spoke’ regions while no difference was detected in the domain-general ATL ‘hub’. The ATL was ubiquitously involved in a wide range of contexts, irrespectively of task requirements and semantic congruity. This is consistent with patients' deficits that, following ATL damage, they show a wholesale decline in the ability to recognise *all* categories of visual items (as compared to other brain areas, whose lesion selectively affects certain categories; see Behrmann et al., 2016). It also fits its hypothesised role as a domain-general apparatus of semantic cognition (Patterson and Lambon Ralph, 2015) and in high-level visual cognition (Clarke and Tyler, 2015). Critically, our data further suggest that the domain-specific ‘spoke’ regions are additionally recruited to enhance processing for semantic congruency about actions and places. Unlike the omnipresence of ATL hub, the spoke regions participated in a content-related fashion: In Experiment 1, extensive clusters of the frontoparietal action system (the pre- and post-central gyri, inferior frontal gyrus, and parietal lobules) were modulated by semantic congruity about actions. These areas correspond to the typical lesion sites seen in apraxia patients (Binkofski and Buxbaum, 2013) and the voluminous fMRI literature about motor imagery and action knowledge (e.g., El-Sourani et al., 2018; Hétu et al., 2013; van Elk et al., 2014; Wurm and Lingnau, 2015). By comparison, in Experiment 2, two key regions of the medial place-related system, the LG and RSC, were recruited to process semantic congruency about places. The LG is known to be the caudal part of the parahippocampal place area, sometimes dubbed ‘lingual landmark area’ (Aguirre, Zarahn, & D'esposito, 1998), and is sensitive to the perceptual integration between foreground and background (Goh et al., 2007). The RSC is crucial for processing the mnemonic features of scenes, such as one's familiarity with a place (Epstein et al., 2007), the viewpoint-invariant identity of a place (Park and Chun, 2009), and the linguistic labels of places (Auger and Maguire, 2018). Recent evidence further shows that the RSC possesses neural patterns that allow the decoding of places from imagined episodes (Robin et al., 2018), and that the RSC favours reminiscing about places over perceiving places (Silson et al., 2019). Taken together, the effects we observed in these action- and place-related spoke regions highlight the fact that the ATL alone is insufficient in dealing with a plethora of detailed semantic information inherent in our environment. To tackle semantic complexity, the hub is supplemented by the spokes so that comprehending nuanced differences becomes possible.

Our connectivity analyses revealed not only the flexibility in the coupling between the hub and spokes, but also the reliable relationship between neural coupling and behavioural outcome. Specifically, the PPI analysis revealed the correspondence between brain and behaviour that explained the variation *between* individuals – we found that the size of behavioural congruency effect significantly modulated the connectivity strength between hub and spokes, with individuals showing a bigger congruency effect also exhibiting greater neural coupling. Furthermore, this correspondence also occurred at the *within*-individual level that explained the ebbs and flows of behavioural effects across runs of scanning. Specifically, the DCM analysis showed that, when the ATL hub intensified its regulatory impact on the spoke, the magnitude of congruency effect tended to increase (i.e., greater hub-to-spoke modulatory impact during a semantically incongruent context corresponded with more decline in accuracy or slower response latency). We speculate that this correspondence might offer important clues about how semantic congruity is neurally implemented in the brain – when confronted with a semantic anomaly (namely, incongruous stimuli, such as a leopard in a kitchen), the ATL hub might enhance its communication with relevant spokes to obtain useful knowledge for processing such anomalous information. However, applying prior knowledge would be unhelpful for solving the task when the stimuli were incongruent. Therefore, ironically, the more intensely the ATL speaks to the spoke, the more deterioration in the performance under incongruent contexts (a drop in accuracy or prolonged reaction), hence a bigger behavioural congruency effect. Future research is needed to test our speculation and elucidate the mechanisms.

## 5 Conclusion

In the present study, we demonstrated that efficient performance on semantic tasks hinges on the neuro-dynamics between the ATL hub and functionally specific spoke regions. Our novel findings unite the contemporary theories of semantic cognition (Lambon Ralph, 2014; Lambon Ralph et al., 2017; Patterson et al., 2007) with existing models for action affordance (van Elk et al., 2014), scene perception (Aminoff, 2014; Epstein, 2014), and embodied cognition (Binder et al., 2016), providing a more comprehensive picture of the mappings between brain and behaviour in a wide range of contexts. Our study provides striking evidence that the anterior temporal lobe is a critical nexus in the neural architecture of action and place knowledge, tying object recognition with functionally-specialised systems. This suggests a direction for future research to look beyond the typical domain-specific regions and consider the contribution of domain-general areas in underpinning conceptual knowledge.

## Footnotes

1 We used a block design to optimise detection of functional connectivity (particularly for the PPI). However, a ‘blocked’ mode of contiguous presentation could make the task less challenging, compared to an event-related design that randomly interleaves different trials (but the randomisation could hinder detection of neural connectivity). This could explain why the congruency effects we observed in accuracy were small, due to the use of a block design. In previous studies (e.g., Joubert et al., 2008; Rémy et al., 2014) wherein the authors used the *same* stimuli as those we used in the present study, the congruent and incongruent stimuli were mixed and presented briefly (e.g., merely 26 ms in Joubert et al., 2008), making the task massively challenging and resulting in a bigger effect (∼7% difference in accuracy).
